# Seasonal population density and winter survival strategies of endangered Kashmir gray langur (*Semnopithecus ajax*) in Dachigam National Park, Kashmir, India

**DOI:** 10.1186/s40064-015-1366-z

**Published:** 2015-09-29

**Authors:** Zaffar Rais Mir, Athar Noor, Bilal Habib, Gopi Govindan Veeraswami

**Affiliations:** Wildlife Institute of India, Chandrabani, P.O Box No.18, Dehradun, 248001 India

**Keywords:** Kashmir gray langur, Population density, Activity budget, Dachigam National Park, Jammu and Kashmir

## Abstract

The population density of Kashmir gray langurs (*Semnopithecus ajax*) was studied in Dachigam National Park (DNP), Kashmir using distance sampling method. A total of 13 transects (1.5–2.5 km in length) were surveyed in the intensive study area (~90 km^2^) yielding 170 encounters in different seasons of the study period (2011–2013). Some aspects of behavior and feeding were also studied during the winter months (Dec–Feb) of 2012 and 2013 inside DNP. We used instantaneous scan sampling to collect behavioral data determining the time budget and diet of langurs in winter conditions. Results suggested that the density of Kashmir gray langurs varied marginally across seasons, with the highest density recorded during winter and lowest during summer season. Langurs spent most of their time in carrying out various social activities (34.32 %) and least in resting (18.41 %). Langurs fed upon 13 plant species (belonging to 12 families) and consumed a substantial proportion of bark (37.4 %) in their diet. We conclude that langur density is low in DNP as compared to other plain areas of the Indian subcontinent and langurs in DNP have balanced their time budget and diet so as to increase their chances of survival in the unfavorably cold and food scarce winter conditions.

## Background

Spatiotemporal variation in food availability has pronounced impacts on population density, dietary patterns and activity budget of animals. In primates, food distribution and abundance affects competitive regimes which in turn may influence the patterns of social relationships, particularly the distribution of affiliative behaviors (Isbell and Young 2002). Studying activity budget and the diet of animals facing harsh conditions offers an insight into their interaction with the environment and their strategies for maximizing energetic and reproductive success.

India is known to harbour about 15 species and 39 sub-species of non-human primates distributed all over, from temperate Himalayan forests in the north to tropical forests in the south. Langurs belong to the most important constituents of the food chain in many Indian forest ecosystems and are preferred prey species for large carnivores such as tiger and leopard (Karanth and Sanquist 1995). The Kashmir gray langur (*Semnopithecus ajax* Pocock, 1928), an old world leaf-eating primate is distributed in fragments along some parts of Pakistan, Nepal and India (Minhas et al. 2010). In India, Kashmir gray langur is distributed in the states of Jammu and Kashmir and Himachal Pradesh (Roberts 1997; Walker and Molur 2004). Present threats to this species mainly include agriculture and development practices (Nowak 1999; Bagchi et al. 2003; Molur et al. 2003). Thus, the International Union for the Conservation of Nature and Natural Resources (IUCN) in 2008 classified ‘*Semnopithecus ajax*’ as “Endangered” (IUCN 2012). Most of the information available about the ecology and biology of gray langurs is by virtue of the Hanuman langur (*Semnopithecus entellus* Dufresne, 1797) either directly studied as a generic langur species or as a subspecies which later got the status of a separate species (Jay 1965; Sugiyama 1965; Vogel 1971; Mohnot 1974; Roonwal and Mohnot 1977; Rajpurohit 1987; Srivastava 1989; Bennett and Davies 1994; Chalise 1995; Schuelke 1998; Chhangani 2000). A few ecological studies have been conducted on gray langur in Himalayan regions (Sugiyama 1976; Sayers and Norconk 2008; Sayers et al. 2010; Minhas et al. 2010, 2012, 2013) but no systematic study has been carried out on Kashmir gray langur in the state of Jammu and Kashmir, India. Wildlife research in this region has taken a back seat owing to more than two decades of political instability.

Kashmir gray langurs are mainly folivorous, but also consume fruits, flowers, cultivated crops, seeds with high levels of toxins, like, strychnine and distasteful vegetation usually avoided by other animals (Minhas et al. 2010). They are flexible in their habitat choice and, correspondingly, are found in wide range of habitat types (Sugiyama 1976; Oppenheimer 1977; Bennett and Davies 1994). Since this species is endemic to Himalayan ecosystems, it has to face harsh climatic conditions during winter and overcome various challenges, like scarce food resources, the seasonal increase in daily energy requirements, higher frequency of extreme weather events like snowfall and shortened day length. They are expected to adopt behavioral strategies aiming at maintaining their energy balance under such variable environmental conditions. In food scarce conditions primates can opt for either of two strategies: (1) they increase travel distance or invest more time in searching for preferred foods, this is however energetically expensive process (Sayers and Norconk 2008; Harris et al. 2010), (2) they decrease their distance moved and modify their diet spectrum to less preferred easily available food items (Ganas and Robbins 2005; Fan et al. 2012). In winter, the first option is not available for primates living in temperate ecosystems as profitable food is scarce or absent. So we hypothesized that langurs in DNP feed upon substantial proportion of non-profitable food items during winter. Further, they must be devoting comparatively more time to foraging than to resting in order to overcome energetic stress and exploit the available resources profitably. This study was conducted to test the above hypothesis while giving an account of seasonal density, time budget and feeding of the Kashmir gray langurs during the resource crunch winter months in the Himalayan ecosystem of the Dachigam National Park, India. The results are intended to provide the baseline information for conducting further studies on this important species and help in its long-term conservation.

## Methods

**Fig. 1:**
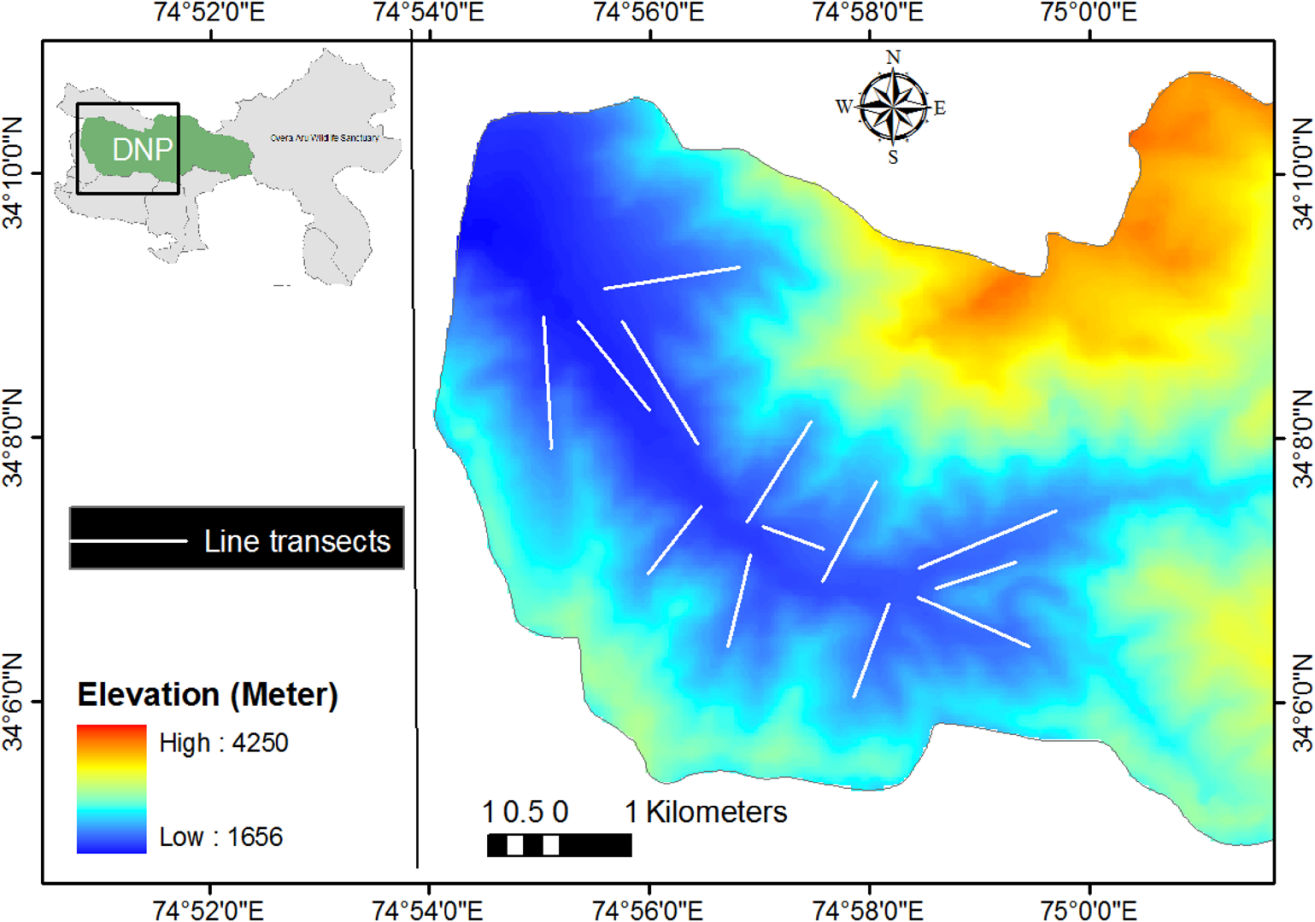
Map showing the digital elevation model of Dachigam National Park along with the locations of line transects

**Fig. 2:**
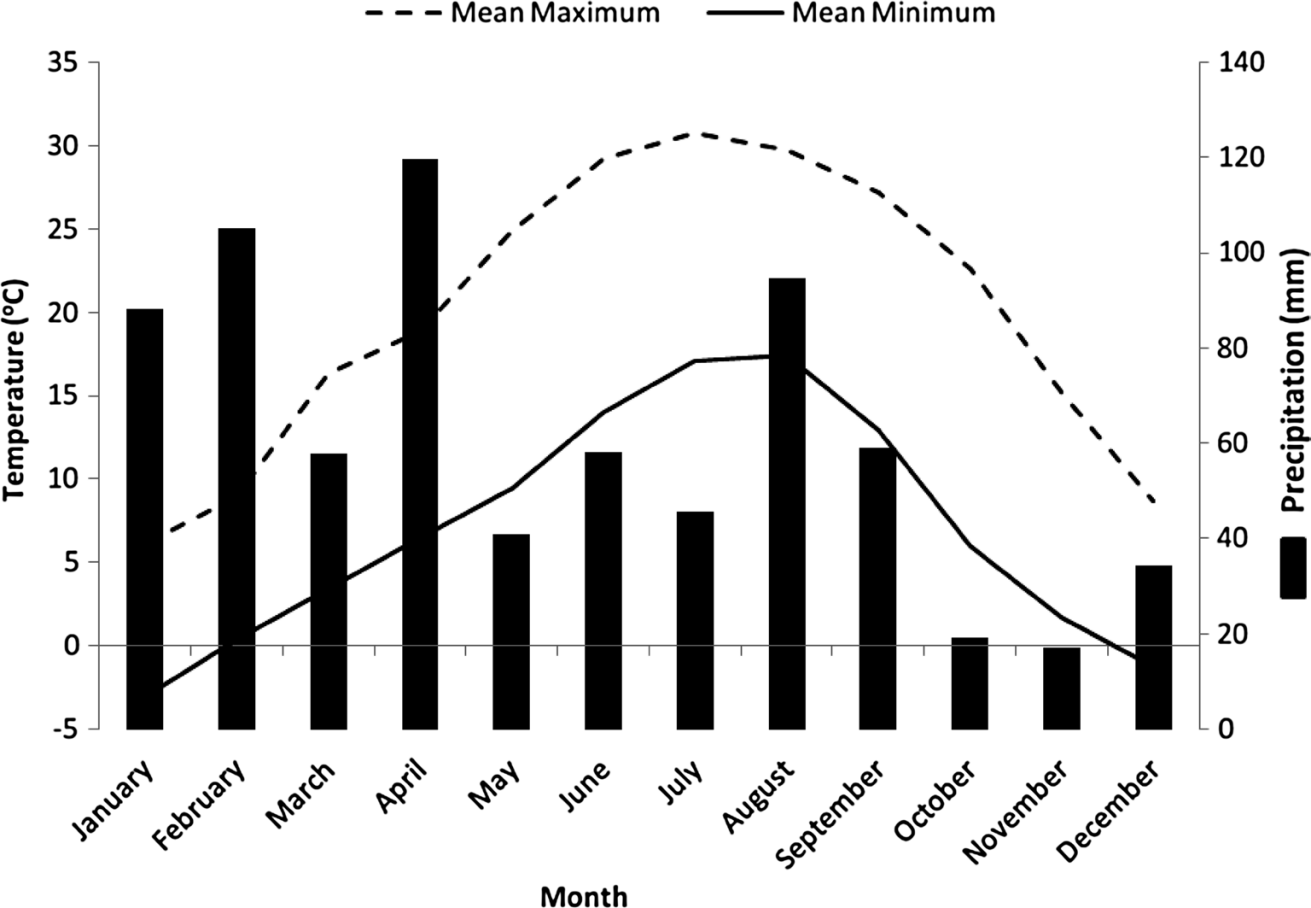
Monthly mean maximum temperature (°C, *dashed line*), monthly mean minimum temperature (°C, *solid line*) and average monthly rainfall (mm, histogram) from 2011 to 2013 recorded at observatory of Sher-e-Kashmir University of Agricultural Sciences and Technology of Kashmir, near Dachigam National Park

The study was conducted in Dachigam National Park (141 km^2^) located between 34°05′–34°12′N and 74°54′–75°09′E in the great Zanskar Range of Himalaya (Fig. 1). The park is the catchment area of the famous Dal lake and is divided into Lower Dachigam in the west and Upper Dachigam in the east. We selected lower Dachigam (~90 km^2^) as intensive study area as langur distribution is limited to this area of the park owing to its suitable altitudinal range. DNP is bounded by Dara block of the Sindh Forest Division in the north; by Brain block, Khrew and Tral ranges of Forest Plantation Divisions in the South; by Harwan village in the west; and by Lidder Forest Division in the east. Monthly mean maximum temperatures ranged from 29 °C in summer to 6 °C in winter while as mean minimum temperatures ranged from 17 °C in summer to—3 °C in winter during our study. The minimum and maximum monthly precipitation ranged from 17 to 120 mm (Fig. 2). Vegetation of lower Dachigam is classified as Himalayan Moist Temperate Forest (Champion and Seth 1968). Climax communities are: riverine forest (1600–1800 m); *Morus alba* community (1700–1900 m); blue pine (*Pinus griffithii*) forest (1700–3000 m); Silver fir (*Abies pindrow*) forest (2300–3200 m); birch forest (2900–3700 m); tall evergreen shrub (3200–3400 m); dwarf evergreen shrub (3500–3700 m); and alpine pastures.

### Population density

We estimated the population density of Kashmir gray langurs in DNP on the seasonal basis from 2011 to 2013, using the Distance sampling technique (Burnham et al. 1980; Buckland et al. 1993). In total thirteen line transects (ranging from 1.5 to 2.5 km in length) were laid randomly in different habitats in lower Dachigam (area ~90 km^2^) covering a distance of 26.05 km (Fig. 1). Each transect was walked 12–18 times per season during morning hours. Total transect effort for 3 years (2011–2013) was 1499.1 km. The program DISTANCE (version 6.0; Laake et al. 1998) was used to analyze the data and estimate animal densities. Minimum Akaike information criterion was used to judge the models after pooling the sighting distances into suitable intervals and truncating the farthest 5 % of the observations wherever needed (Buckland et al. 1993). Nonparametric Kruskal–wallis test was used to test for significance in density difference across seasons.

### Activity and diet

Activity and feeding of Kashmir gray langurs was studied during the winter months (Dec–Feb) of 2012 and 2013. Instantaneous scan sampling (Altman 1974; Martin and Bateson 1993) was used to study different activities of langurs. Whenever a group was encountered, individuals were observed cyclically, scanning the entire group from left to right using a field binocular (Nikon, 8 × 42) from a distance ranging from 10 to 25 m. Langurs did not show any fearful behavior from such distance and continued to engage in their daily routine activity as they are used to human disturbances and are sometimes even involved in crop raiding around the park. The scan interval was set at 5 min and with the help of 3–4 observers, each individual’s state was noted instantly and for a brief time. Animals were observed on 73 observation days (168.5 h total observation time). Observation hours usually started at 8:00 h in the morning and ended at 17:00 h or when the group went out of sight. Each observation was subcategorized into different types of behaviors as: moving, resting, foraging and social behaviors. To study feeding behavior, we defined foraging as active intake of food and searching of food items. On each foraging observation, item taken, species of plant and plant part was also recorded. Langur individuals were divided into three groups: male, female and juvenile. For data analysis, we calculated total counts of each behavior noted. The time spent in each behavior was then calculated as a percentage of the total count of all behaviors per class (time budget). The time the langur groups were observed in each time interval (time seen) was calculated as a percentage of the total observation time (168.5 h). Chi square test was used to test the difference in activity budgets of different groups.

### Ethical note

We used non-invasive instantaneous scan sampling for behavioral study, which doesn’t involve capturing or handling of animals. Moreover during our study, we maintained a distance of about 10–25 m from the study animals while observing them and tried to minimize the disturbance caused by our presence.

## Results

### Population status

A total of 170 encounters comprising of 2679 individuals were recorded in 748 transect walks during the entire study. Different groups ranging from one male individual to 103 individual multi-male multi-female groups were sighted in different habitats. The results from line transects intended to provide seasonal density of Kashmir gray langur. There was no significant difference in the density of langurs across different seasons (Kruskal–Wallis Test, H = 3.0, df = 3, p = 0.39), although highest density was recorded in winter season (22.05 ± 5.12 individuals/km^2^) and lowest in the summer season (09.35 ± 3.03 individuals/km^2^). Average group density varied from 0.50 ± 0.13 groups/km^2^ to 1.21 ± 0.18 groups/km^2^. Average group size of langurs did not show much variation across seasons. The population density, troop size, group density, effective strip width and other important parameters of Kashmir gray langur recorded in different seasons are given in Table 1.

**Table 1:** Season wise individual and group density of Kashmir gray langur in Dachigam National Park, India

Parameters	Winter	Spring	Summer	Autumn	Pooled
Individual density (no. of animals/km^2^)	22.05	20.48	9.35	13.29	16.32
Standard error	5.12	3.67	3.03	3.35	1.87
Percent CV	23.22	17.96	32.47	25.27	11.49
Group density (no. of Gps/km^2^)	1.21	1.3	0.5	1.06	0.99
Standard error	0.18	0.19	0.13	0.24	0.08
Percent CV	14.94	14.81	27.82	23.2	8.97
Effective strip width (m)	72.42	53.34	54.98	42.37	56.81
Standard error	7.42	5.26	10.81	6.75	3.35
Percent CV	10.26	9.87	19.68	15.95	5.91
Average group size	18.154	15.66	18.65	12.44	16.43
Standard error	3.22	1.59	3.12	1.24	1.17
Encounter rate (no. seen/km walk)	0.17	0.13	0.05	0.09	0.11
Percent CV	10.86	11.04	19.67	16.85	6.76
Detection probability	0.45	0.44	0.36	0.32	0.28

### Time budget

**Fig. 3:**
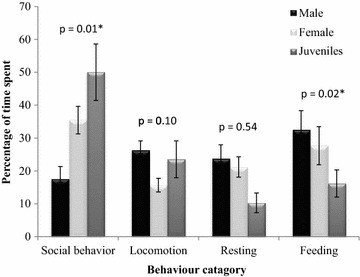
Daily time budget (mean ± SE) of Kashmir gray langur in Dachigam National Park during winter months of year 2012 and 2013

In winter season, the Kashmir langur spent an average 34.32 % of their time in carrying out various social activities like grooming, playing and agonistic activities, followed by time spent on foraging (25.44 %), moving (21.81 %), and the least time was spent in resting which accounted for 18.41 % of their daily activities. Chi square test revealed significant difference in the amount of time spent by male, female and juvenile langurs in social activities (χ^2^ = 8.27, df = 2, p = 0.01) and feeding (χ^2^ = 7.19, df = 2, p = 0.02) while there was no significant difference in time spent in resting (χ^2^ = 1.20, df = 2, p = 0.54) and moving (χ^2^ = 4.42, df = 2, p = 0.10) (Fig. 3). Male individuals spent maximum time feeding (32.50 %) followed by moving (26.25 %), resting (23.75 %) and social behavior (17.50 %) while female individuals spent 35.48 % of daily time in various social activities followed by foraging activity (27.64 %), resting (21.19 %) and moving (15.66 %). Juveniles were recorded to spent maximum (50 %) of their day time carrying out various social activities, which included playing, followed by locomotion (23.52 %), feeding (16.17 %) and resting (10.29 %).

### Feeding

**Fig. 4:**
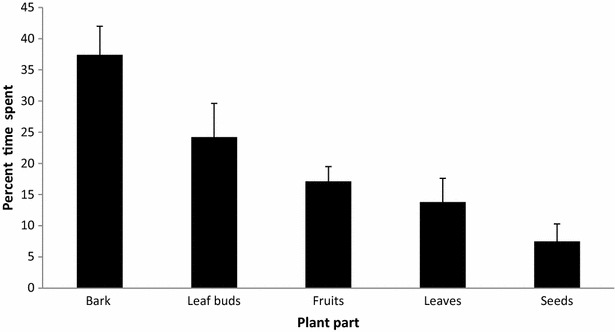
Percentage of time spent (mean ± SE) feeding on different plant parts by langur during winter months (2012 and 2013) in Dachigam National Park

Langurs were observed to feed upon 13 plant species found naturally in their habitat. This included consumption of various plant parts such as bark, leaf buds, fruit, leaves, and seeds that varied with species consumed. Bark constituted 37.4 % of their diet followed by leaf buds (24.2 %), fruits (17.1 %), leaves (13.8 %) and seeds (7.5 %) (Fig. 4). Also, more than 50 % of langur diet was found to be made up by *Aesculus indica*, *Ulmus wallichiana* and *Quercus robur* during the winter season. The percentage contribution of other species to the langur diet is given in Table 2. Langurs were also observed to feed occasionally on body lice (*Pediculus* spp) picked up from the bodies of the other individuals during grooming and other insects obtained from bark removal or stone turning.

**Table 2:** Plants consumed by langur in winter and their rate of consumption in Dachigam National Park, India

S. no.	Name of plant consumed	Local name	Family	Type	Part consumed	Rate of consumption (%)
1	*Aesculus indica*	Han doon	Sapindaceae	Tree	Buds, bark, fruit, seeds.	23.35
2	*Ulmus wallichiana*	Bren	Ulmaceae	Tree	Bark	21.11
3	*Quercus robur*	Oak	Fagaceae	Tree	Bark, buds, fruit	18.19
4	*Populus ciliata*	Fras	Salicaceae	Tree	Bark	9.78
5	*Salix sp.*	Vir	Salicaceae	Tree	Bark, buds	8.96
6	*Hedera nepalensis*	Kateembri	Araliaceae	Climber	Leaves	5.43
7	*Robinia pseudoacacia*	Kikar	Fabaceae	Tree	Bark, buds	4.07
8	*Pinus wallichiana*	Kail	Pinaceae	Tree	Shoots, seeds, bark	2.72
9	*Morus sp.*	Tul	Moraceae	Tree	Buds	2.04
10	*Parrotiopsis jacquemontiana*	Hatab	Hamamelidaceae	Tree	Bud, shoots	1.70
11	*Celtis australis*	Brimij	Cannabaceae	Tree	Bark, seed	1.36
12	*Prunus sp.*	Gordol	Rosaceae	Tree	Bark, buds	0.68
13	*Ziziphus mauritiana*	Brey	Rhamnaceae	Tree	Bark, buds	0.61

## Discussion and conclusion

The present study determines the population density of Kashmir gray langur in DNP across different seasons. Further it provides an insight on adaptations of langur in the resource crunch period of winter. The hypothesis that langurs in DNP feed upon substantial proportion of non-profitable food items during winter was proven to be correct as the results clearly indicated high dependence of langurs on tree bark in winter months. Moreover we predicted that, they must be devoting more time to foraging compared to resting and the results of this study are in agreement with this, as the langurs spent least time in resting and the second highest time was spent on foraging activity. In addition to food dearth in winter, langur being one of the preferred preys of leopard in DNP has to face high predation pressure (Habib et al. 2014). In this study, the overall population density of langurs in DNP was calculated as 16.32 individuals/km^2^ which is almost same as the density of Kashmir gray langurs (16.01/km^2^) reported from similar Himalayan ecosystem of Machiara National Park, Pakistan (Minhas et al. 2012). Density of langurs did not vary significantly across different seasons, only a marginal fluctuation in density was observed which can be attributed to the movement of langurs from upper areas including some adjacent protected areas towards lower comparatively less hostile areas of DNP in the winter months. Minhas et al. (2012) has also reported fluctuation in langur population in Machiara NP due to the migration of troops. Genus *Semnopithecus* has been studied extensively in the Indian subcontinent, especially the species *Semnopithecus entellus* (Hanuman langur or gray langur). The density of langurs in our study was lower than densities of gray langurs reported from other food abundant habitats of the country. Bagchi et al. (2003) estimated 21.7 individuals/km^2^ in Ranthambore, Edgaonkar (2008) reported 28.3 individuals/km^2^ in Bori-Satpura tiger reserve, Narasimmarajan et al. (2012) reported 42.92 individuals/km^2^ from Melghat Tiger Reserve, Maharashtra and the highest density of 82.5 individuals/km^2^ was estimated in Pench tiger reserve by Majumder et al. (2010).

The time budgets of the folivorous colobines are largely influenced by their diet (Schneider et al. 2010). So food availability has a crucial role to determine time budget of these primates. They usually spend majority of their time resting and considerably less time feeding and moving (Clutton-Brock 1977; Stanford 1991; Fleagle 1999), but in case of this study, they spent relatively more time in carrying out social activities followed by foraging and then the locomotion. However, least time was allocated to resting as it was hypothesized earlier that they may sacrifice their resting time to increase foraging effort. A clear reason behind this is scarcity of food due to heavy snow cover during winter. Feeding time in winter may also be high due to more energy consumption for thermoregulation at lower temperatures (Hill 2006). And they might be on negative energy budget during the winter thereby requiring food more often, and thus spend more time foraging. There are other studies also which have concluded that primates respond to snow coverage and low temperature by maximizing feeding efficiency and attempting to ingest more food (van Doorn et al. 2010; Majolo et al. 2013). Increased feeding time as a response to low food availability has been documented for various primates living in temperate regions (Guo et al. 2007; Sayers and Norconk 2008; Mendiratta et al. 2009; Sayers et al. 2010). It was found that juveniles spent considerably more time in social behaviors than adults. The same is true for the white-headed langur (*Trachypithecus poliocephalus*) (Li and Rogers 2004), and is associated with their physical and behavioral development and socialization (Poirier et al. 1978).

In this study, langurs were seen feeding upon 13 species of plants belonging to 12 families. Gray langurs are mainly classified as folivorous species, as they have multi-chambered stomach, specialized for the digestion of leaves (Amerasinghe et al. 1971; Minhas et al. 2010). We tested the prediction that langurs relied highly upon low quality diet in food scarce conditions of winter in order to avoid energy crises. And it was found true as tree bark constituted of 37.4 % diet of langurs in DNP. This high dependence upon bark clearly shows their flexibility in food preference. As there are no or fewer green leaves available in DNP in winter months, they shift their diet to low quality non-folivorous diet comprising mainly of bark. Sayers and Norconk (2008) also emphasized on their ecological generalist behavior and reported similar results from Nepal where leaf buds comprised a major portion of the Himalayan gray langur diet in winter, particularly from *Cotoneaster frigidus* and *Sorbus cuspidata,* and ripe fruit, e.g., *Berberis aristata* and *C. frigidus*. They also reported langurs feeding upon bark from more than five woody plants in winter season, which usually was avoided in other seasons. Apart from acting as a substitute for preferred folivorous diet in the lean season, bark helps to increase the health quality of primates by providing other benefits. Vuorela (2005) studied the bark of *Pinus pinaster* and found it as a rich source of pro-cyanidin oligomers, which are bioactive sources of plant phenolics. These are effective against the formation of the pro-inflammatory mediator prostaglandin (E2). Vuorela (2005) concluded that pine bark phenolic extracts are safe and bioactive for possible food applications including functional foods intended for health benefits. They were observed to feed upon the only available fruits, of *Aesculusindica* and *Quercusrobur*. *A.indica* constituted about (23.35 %) of langur’s diet; they were seen feeding upon its bark and leaf buds as well. The seeds of *A.indica* are known to have high nutritional value containing good amounts of various mineral elements (Majeed et al. 2010). The nutritional value of *Quercus sp* is also known to be high containing larger quantities of nutrients per kg of biomass than other commercial forest species in the region, such as Pinus (Miguel et al. 2006). Hence, both these plant species (*A.indica* and *Q.robur*) were consumed in the high proportion as they provide a good dietary supplement to langurs in food scarce conditions of winter.

An understanding of density and population size is, of course, a key factor in future contingencies for the effective management and conservation of the species. However, further research is needed to understand the habitat requirements and potential threats that this endangered species is facing in this area. Since, overall density of langurs was found low in our study, the conservation of this langur population should become a priority now, to avoid decline in population of this endemic primate. Activity and feeding observations made in our study highlighted the generalist nature of Kashmir gray langur and focused on behavioral as well as dietary adaptations opted by langurs to thrive in a food scarce and climatically hostile Himalayan ecosystem. Physiological approaches need to be used in the future studies to measure energetic and climatic stress faced by Kashmir gray langurs in temperate conditions of Himalaya. Our data can serve as a management tool to increase the base of existing food resources in and around DNP to support the langur population in the park and make their long-term conservation possible. Further, this study can become a base for future studies on the species in this part of Himalaya.
